# Hysterectomy and gallstone risk in women: A cross-sectional NHANES study on the mediating role of metabolic disorders

**DOI:** 10.1097/MD.0000000000051413

**Published:** 2026-10-30

**Authors:** Xiaolin Li, Lang Song

**Affiliations:** a Neijiang Hospital of Traditional Chinese Medicine, Neijiang, China; b Department of Gastrocolorectal Surgery, The Second Hospital of Shanxi Medical University, Taiyuan, Shanxi, China.

**Keywords:** BMI, diabetes, gallstones, hypertension, hysterectomy

## Abstract

Hysterectomy is a common gynecological procedure, with growing evidence suggesting potential long-term metabolic consequences. Gallstone disease (GSD) is a prevalent condition strongly linked to metabolic disturbances. However, the direct association between hysterectomy and GSD, and the potential mediating role of metabolic disorders, remains inadequately explored. This cross-sectional study analyzed data from 5317 female participants (aged ≥ 20) in the National Health and Nutrition Examination Survey cycles 2017 to 2023. The association between self-reported hysterectomy and GSD was assessed using weighted multivariable logistic regression, adjusting for sociodemographic, lifestyle, and clinical factors. Subgroup and E-value analyses evaluated heterogeneity and robustness. Mediation analysis quantified the contributions of body mass index, hypertension, hyperlipidemia, and diabetes. A history of hysterectomy was significantly associated with increased odds of GSD (fully adjusted odds ratio = 1.32, 95% confidence interval: 1.10–1.59). The association was more pronounced in women with lower socioeconomic status. Mediation analysis revealed that body mass index (proportion mediated: 16.5%), diabetes (13.9%), and hypertension (6.5%) were significant mediators, whereas hyperlipidemia was not. Hysterectomy is independently associated with a higher prevalence of gallstones in Unites States women. This association is partially mediated through metabolic pathways, particularly obesity and diabetes. These findings highlight the importance of postoperative metabolic monitoring and management for women undergoing hysterectomy to mitigate long-term GSD risk.

## 1. Introduction

Hysterectomy is one of the most common gynecological procedures performed globally, with a substantial number of women undergoing this surgery for benign conditions annually.^[1]^ Despite advancements in surgical techniques, accumulating evidence suggests that hysterectomy may exert extensive metabolic effects on women’s long-term health beyond the pelvis.^[2]^ Multiple studies have revealed that hysterectomy, even with ovarian conservation, is significantly associated with an increased risk of metabolic syndrome and may independently elevate the risk of cardiovascular disease and stroke.^[3,4]^ The underlying biological mechanisms may involve surgery-induced alterations in the hormonal milieu, activation of inflammatory states, and disruption of metabolic homeostasis.^[5]^ Postoperative weight gain is a frequently observed phenomenon, and further research has identified associations between hysterectomy and elevated blood glucose levels as well as an increased risk of hypertension. Notably, these very metabolic shifts (weight gain, hyperglycemia, hypertension) mirror the key drivers of biliary cholesterol supersaturation, offering a plausible mechanistic backdrop to the frequent intraoperative detection of occult gallstones during hysterectomy workups.^[6–8]^ Hysterectomy may also impact liver health, being linked to a higher prevalence of nonalcoholic fatty liver disease.^[9]^ Collectively, this evidence points to hysterectomy as a significant metabolic event that may reshape women’s long-term health trajectories.

On the other hand, gallstone disease (GSD) is a highly prevalent gastrointestinal disorder, affecting approximately 10–20% of the global adult population.^[10]^ Its formation is not an isolated event but is closely related to metabolic disturbances. Substantial research confirms that GSD shares core pathophysiological foundations with metabolic syndrome, including insulin resistance, obesity, and dyslipidemia.^[11]^ While genetic factors play a significant role, acquired metabolic abnormalities are key promoters of stone formation.^[10]^ Crucially, these acquired metabolic disturbances, rather than genetic predisposition, are modifiable, making them the most plausible intermediate through which hysterectomy could exert its effect on gallstone risk. Clinically, gynecologists frequently discover concurrent asymptomatic gallstones during preoperative evaluations for hysterectomy, raising considerations for simultaneous cholecystectomy.^[12]^ This observation indirectly suggests that the 2 conditions may share common underlying risk factors or pathological links.

However, current research predominantly focuses on the impact of hysterectomy on cardiometabolic endpoints or the metabolic etiology of GSD itself.^[4,11]^ There is a lack of in-depth investigation into whether and how hysterectomy specifically influences the risk of developing gallstones, and the mechanisms underlying this association remain underexplored. Based on this background, we propose the scientific hypothesis that hysterectomy may increase the risk of GSD by inducing or exacerbating a series of intermediary metabolic abnormalities, such as obesity, hypertension, hyperlipidemia, and diabetes. In other words, these metabolic factors may play a key mediating role in the association between “hysterectomy” and “gallstones.”

To test this hypothesis, this study utilizes the nationally representative National Health and Nutrition Examination Survey (NHANES) database to conduct a cross-sectional analysis. Our primary aim is to elucidate the epidemiological association between hysterectomy and gallstone prevalence. Furthermore, we intend to employ mediation analysis models to quantitatively assess the mediating effects of body mass index (BMI), hypertension, hyperlipidemia, and diabetes in this relationship. The findings of this study are expected to provide a novel metabolic perspective for understanding the long-term health impacts of hysterectomy and offer potential evidence-based insights for the early identification and targeted metabolic management of women at high risk for gallstones.

## 2. Methods

### 2.1. Data source and study population

This cross-sectional study utilized data from 2 consecutive cycles (2017–2020 and 2021–2023) of the NHANES. Given that the present analysis relied exclusively on deidentified, publicly accessible NHANES datasets, no further review by an institutional ethics committee was deemed necessary. The initial sample comprised 27,493 participants. After excluding males (n = 13,296), individuals aged < 20 years (n = 5123), and those with missing data on the primary exposure (hysterectomy) or outcome (gallstones) variables (n = 1757), the sample was reduced to 6860. Further exclusion of participants with missing data on any covariate yielded a final analytic sample of 5317 women, including 782 with gallstones and 4535 without (Fig. 1).

**Figure 1.**
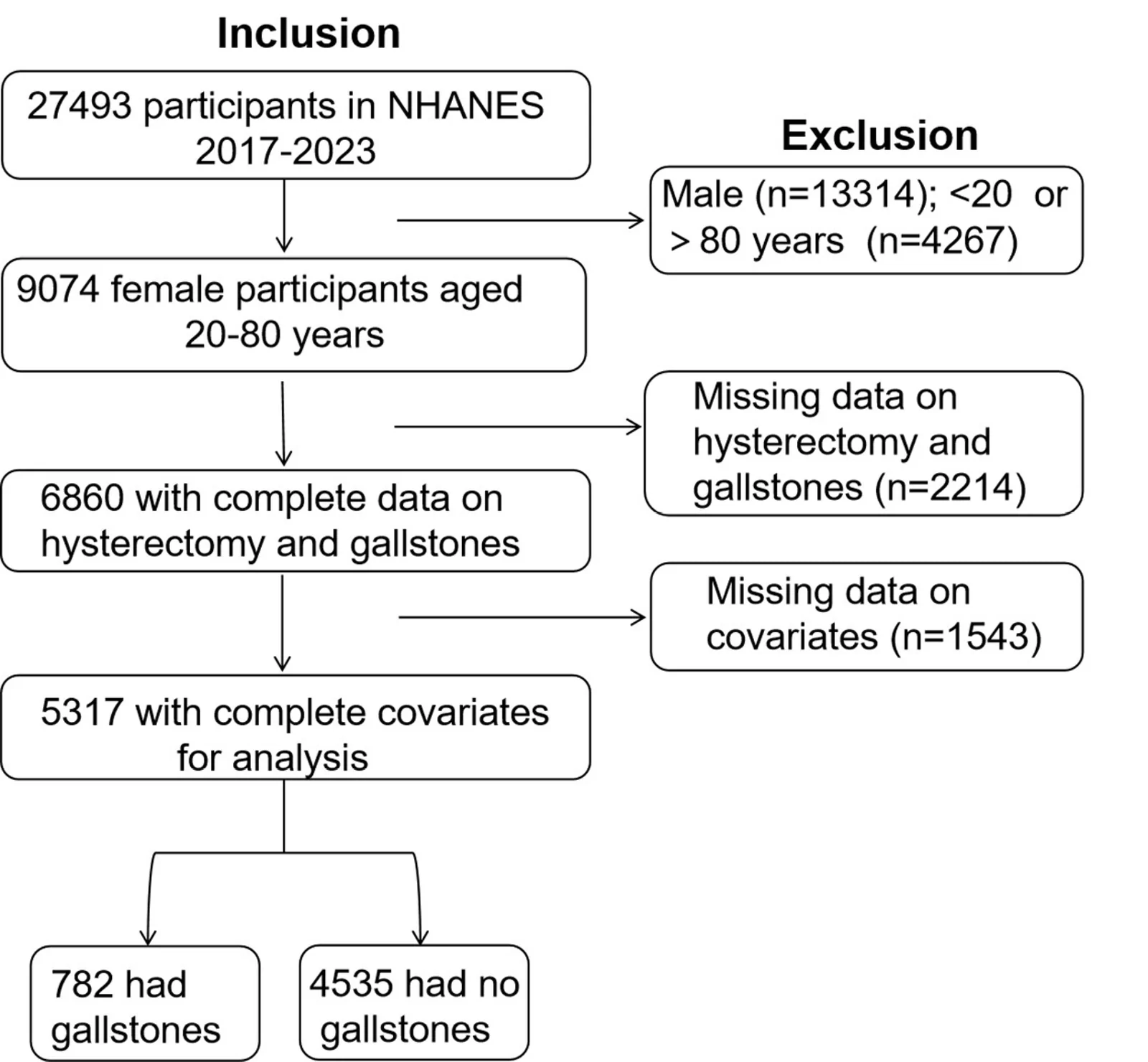
Flowchart of the study. n = number of participants, NHANES = National Health and Nutrition Examination Survey.

### 2.2. Ascertainment of primary variables

The core independent variable, hysterectomy status, was derived from the questionnaire item: “Have you/Has sample person had a hysterectomy that is, surgery to remove your/her uterus or womb?” Responses were categorized as “Yes” or “No.”

The outcome variable, GSD, was based on the question: “Has a doctor or other health professional ever told you/sample person that you/she/he had gallstones?” Responses were categorized as “Yes” or “No.”

### 2.3. Covariates

Multiple covariates confirmed to be associated with both hysterectomy and gallstone risk were included in multivariable logistic regression models to control for potential confounding effects. These included sociodemographic factors (age, race/ethnicity, educational attainment, marital status, and family poverty–income ratio [PIR]) and lifestyle variables (BMI, smoking status, and alcohol use). Considering that metabolic diseases (diabetes, hypertension, and hyperlipidemia) might act as important mediators or confounders in the relationship between hysterectomy and gallstones, these variables were also adjusted for.^[13]^ All categorical variables were constructed following clinical conventions. Hypertension and diabetes were defined based on self-reported physician diagnosis. Hyperlipidemia was defined according to the adult treatment panel III (ATP III) of the National Cholesterol Education Program criteria, which include low-density lipoprotein cholesterol ≥ 130 mg/dL, total cholesterol ≥ 200 mg/dL, triglycerides (TGs) ≥ 150 mg/dL, or high-density lipoprotein cholesterol ≤ 50 mg/dL for women and ≤ 40 mg/dL for men.^[14]^

### 2.4. Descriptive analysis

Data analyses accounted for the complex sampling design of NHANES, incorporating clustering, stratification, and sampling weights to ensure national representativeness. Descriptive analyses included demographic characteristics, socioeconomic status, lifestyle factors, and clinical indicators. Categorical variables are presented as unweighted counts and weighted percentages. Continuous variables are presented as unweighted mean ± standard deviation (with weighted mean in parentheses). Between-group comparisons were performed using weighted chi-square tests for categorical variables and weighted *t*-tests for continuous variables, with statistical significance set at *P* < .05. All statistical analyses were performed using R software (version 4.3.1; R Foundation for Statistical Computing) with the survey package.

### 2.5. Multivariable logistic regression analysis

Multivariable logistic regression was employed to explore the association between hysterectomy and gallstones. Four hierarchical models were constructed. The Crude model (Model 1) included only hysterectomy as the independent variable without any covariate adjustment. Model 2 adjusted for demographic characteristics: age, race/ethnicity, education level, marital status, and PIR. Model 3 further adjusted for lifestyle-related variables: BMI, smoking status, and alcohol use. Model 4, the fully adjusted model, additionally included clinical variables: diabetes, hypertension, and hyperlipidemia. All analyses used weighted logistic regression models. Odds ratios (ORs) and their 95% confidence intervals (CIs) were calculated for each variable. Statistical significance was set at a 2-tailed *P* value < .05. Model fit was assessed using akaike information criterion and McFadden’s R^2^.

### 2.6. Subgroup analysis and E-value analysis

A combination of stratified subgroup analysis and E-value sensitivity analysis was used to systematically evaluate the heterogeneity and robustness of the association between hysterectomy and gallstones. Subgroup analyses were stratified across 8 dimensions: age (≤ 65 vs > 65 years), BMI (< 25 [normal], 25–29.9 [overweight], and ≥ 30 kg/m^2^ [obese]), race/ethnicity (non-Hispanic White, non-Hispanic Black, other races), education level (high school or below vs some college/college graduate or above), marital status (married/living with partner, widowed/divorced/separated, never married), family PIR (< 1.3, 1.3–3.5, > 3.5), smoking status (yes/no), and alcohol use (yes/no). All subgroup analyses were uniformly adjusted for age, race, education, marital status, PIR, BMI, smoking, alcohol use, diabetes, hypertension, and hyperlipidemia. The association strength within each subgroup is presented as OR and 95% CI. The statistical significance of effect modification was tested by calculating the *P* value for interaction between each stratification variable and hysterectomy by including an interaction term in the multivariable logistic regression model.

Furthermore, E-value analysis was employed to assess the potential impact of unmeasured confounding. Based on the results from the fully adjusted multivariable logistic regression model, the E-value quantifies the minimum strength of association that an unmeasured confounder would need to have with both the exposure (hysterectomy) and the outcome (gallstones) to fully explain away the observed association or to shift the CI to include the null value.

### 2.7. Mediation analysis

Mediation analysis was conducted to assess the roles of BMI, hyperlipidemia, hypertension, and diabetes in the hysterectomy–gallstone association, using the difference method on the log-odds scale. Let c denote the total log-OR of hysterectomy on gallstones, and c′the direct log-OR after adjusting for the mediator; the indirect effect = c − c′, and the proportion mediated = (c − c′)/c × 100%. The a-path (hysterectomy → mediator) and b-path (mediator → gallstones) were estimated via weighted logistic regression adjusting for age, race/ethnicity, education, marital status, PIR, smoking, and alcohol use. All indirect effects and 95% CIs were obtained using 1000 bootstrap resamples. Notably, these analyses yield statistical mediation effects under the assumed temporal ordering; they do not establish causal mediation, given the cross-sectional design of this study.

### 2.8. Ethics statement

This investigation, utilizing data from the NHANES, was performed in compliance with ethical guidelines approved by the Research Ethics Review Board of the National Center for Health Statistics and the Human Use Review Committee of the United States Army Research Institute of Environmental Medicine. Written informed consent was obtained from every participant prior to their involvement in the study.^[15]^ Further details regarding ethical protocols and standards can be accessed through the official NHANES website (https://www.cdc.gov/nchs/nhanes/).

## 3. Result

### 3.1. Descriptive analysis

Descriptive analysis revealed that the prevalence of hysterectomy was significantly higher in women with gallstones compared to those without (30.2% vs 18.4%, *P* < .0001). Regarding demographic characteristics, women with gallstones were significantly older (57.3 ± 15.1 years vs 50.3 ± 17.2 years, *P* < .0001) and had a higher BMI (34.0 ± 8.7 kg/m^2^ vs 29.8 ± 7.8 kg/m^2^, *P* < .0001). A higher proportion of gallstone patients were non-Hispanic White (71.3% vs 64.9%), while a lower proportion were non-Hispanic Black (6.5% vs 11.3%). In terms of education, gallstone patients had higher proportions of high school graduates (29.8% vs 23.2%) and individuals with some college education (35.5% vs 31.6%), but a lower proportion of college graduates or above (24.7% vs 37.6%). Marital status showed that gallstone patients had a higher proportion of widowed/divorced/separated individuals (28.1% vs 22.7%). Regarding health conditions, the gallstone group had significantly higher prevalence rates of diabetes (18.7% vs 8.9%, *P* < .0001), hypertension (45.0% vs 28.1%, *P* < .0001), and hyperlipidemia (69% vs 62%, *P* < .0001). For lifestyle factors, a higher proportion of smokers was observed in the gallstone group (46.1% vs 32.9%, *P* < .0001), while alcohol use showed no significant difference between groups (91.7% vs 91.8%, *P* = .962). Socioeconomic indicators showed that the gallstone group had a lower family PIR (2.6 ± 1.6 vs 2.8 ± 1.6, *P* = .004) (Table 1).

**Table 1:** Characteristics of study participants by gallstone status.(n = 5317, aged 20–80 years)

Variable	Level	No gallstones, n (%)	Has gallstones, n (%)	*P* value
Hysterectomy	No	3612 (81.6)	521 (69.8)	< .0001
	Yes	923 (18.4)	261 (30.2)	
Race Ethnicity	Mexican American	396 (6.5)	89 (6.9)	.022
	Other Hispanic	435 (7.6)	79 (6.4)	
	Non-Hispanic White	2159 (64.9)	430 (71.3)	
	Non-Hispanic Black	916 (11.3)	106 (6.5)	
	Other Race	629 (9.8)	78 (8.8)	
Education	< 9th grade	184 (2.2)	33 (2.2)	< .0001
	9–11th grade	358 (5.4)	87 (7.8)	
	High school graduate/General Educational Development	899 (23.2)	197 (29.8)	
	Some college or associate degree	1592 (31.6)	295 (35.5)	
	College graduate or above	1502 (37.6)	170 (24.7)	
Marital Status	Married/Living with partner	2378 (59)	433 (60.4)	.001
	Widowed/Divorced/Separated	1250 (22.7)	258 (28.1)	
	Never married	907 (18.4)	91 (11.6)	
Diabetes	No	4013 (91.1)	601 (81.3)	< .0001
	Yes	522 (8.9)	181 (18.7)	
Hypertension	No	3005 (71.9)	402 (55)	< .0001
	Yes	1530 (28.1)	380 (45)	
Hyperlipidemia	No	1661 (38.0)	257 (31.0)	.019
	Yes	2874 (62.0)	525 (69.0)	
Drinking	No	476 (8.2)	75 (8.3)	.962
	Yes	4059 (91.8)	707 (91.7)	
Smoking	No	3006 (67.1)	435 (53.9)	< .0001
	Yes	1529 (32.9)	347 (46.1)	
Age	Mean ± SD (Weighted Mean)	50.3 ± 17.2 (47.9)	57.3 ± 15.1 (56.1)	< .0001
BMI	Mean ± SD (Weighted Mean)	29.8 ± 7.8 (29.4)	34 ± 8.7 (33.8)	< .0001
PIR	Mean ± SD (Weighted Mean)	2.8 ± 1.6 (3.1)	2.6 ± 1.6 (2.9)	.004

Continuous variables are presented as raw mean ± SD (weighted mean in parentheses). Categorical variables are presented as raw n (Weighted, %). *P* values are from weighted chi-square tests for categorical variables and weighted t-tests for continuous variables.

n = number of participants, PIR = poverty–income ratio, SD = standard deviation.

### 3.2. Multivariable logistic regression analysis

Weighted multivariable logistic regression analysis showed a significant positive association between hysterectomy and gallstone risk. In the crude model (Model 1) without any covariate adjustment, women with hysterectomy had 1.96 times the odds of gallstones compared to those without (OR = 1.96, 95% CI: 1.66–2.31, *P* < .001). After adjusting for demographic characteristics (Model 2), the association attenuated but remained significant (OR = 1.40, 95% CI: 1.16–1.68, *P* < .001). Further adjustment for lifestyle factors (Model 3) led to a slight further decrease in association strength (OR = 1.35, 95% CI: 1.12–1.63, *P* = .001). When simultaneously adjusting for demographic, lifestyle, and clinical disease variables (Model 4), the association between hysterectomy and gallstones remained significant (OR = 1.32, 95% CI: 1.10–1.59, *P* = .003) (Table 2).

**Table 2:** Multivariable logistic regression analysis of gallstone.

Comparison	Models	OR (95% CI)	*P* value
Hysterectomy (Yes vs No)	Model 1	1.96 (1.66–2.31)	< .001
	Model 2	1.40 (1.16–1.68)	< .001
	Model 3	1.35 (1.12–1.63)	.001
	Model 4	1.32 (1.10–1.59)	.003

Model specifications.

Model 1: Unadjusted model.

Model 2: Adjusted for age, race/ethnicity, education, marital status, and PIR.

Model 3: Model 2 + BMI, smoking, and alcohol use.

Model 4: Model 3 + diabetes, hypertension, and hyperlipidemia.

BMI = body mass index, CI = confidence interval, OR = odds ratio, PIR = poverty–income ratio.

### 3.3. Subgroup and E-value analysis

Subgroup analysis revealed heterogeneity in this association across populations. The association was more pronounced among older individuals (OR = 1.40 for > 65 years group vs OR = 1.28 for ≤ 65 years group), those with obesity (BMI ≥ 30 group, OR = 1.32), smokers (OR = 1.55), married/cohabiting individuals (OR = 1.43), those with higher education (college/some college group, OR = 1.47), non-Hispanic Whites (OR = 1.33), individuals with low income (PIR < 1.3 group, OR = 1.65), and alcohol users (OR = 1.38). Notably, the PIR showed a significant effect modification (*P* for interaction = .021) (Fig. 2). E-value analysis further assessed the potential influence of unmeasured confounding. The results indicated that an unmeasured confounder would need to be associated with both hysterectomy and gallstones with an OR of 2.01 to fully explain away the observed association, and an OR of 1.58 to shift the 95% CI to include the null value. These thresholds exceed the plausible effect sizes of most known confounding factors, suggesting that the study findings are relatively robust to potential unmeasured confounding.

**Figure 2.**
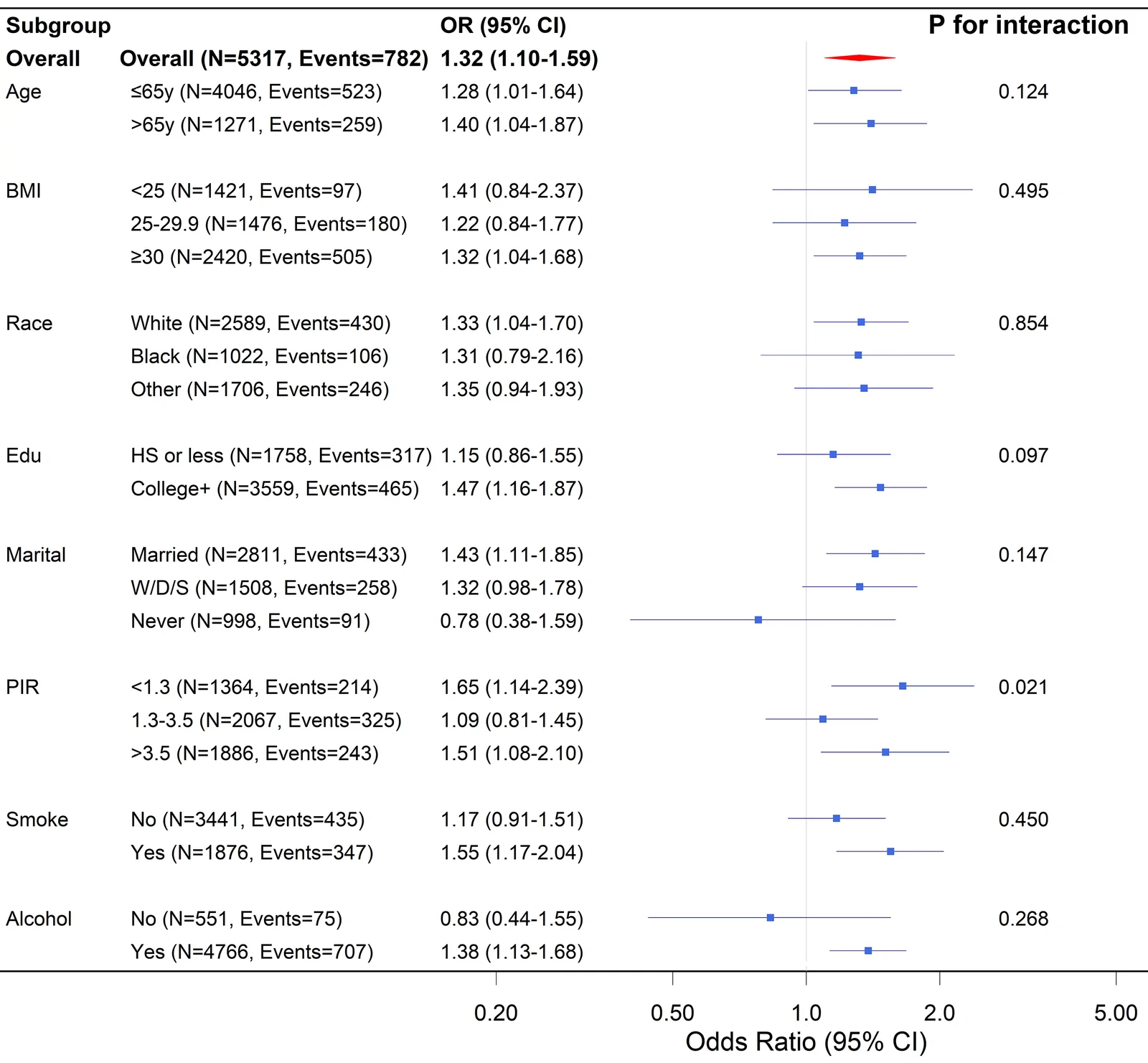
Subgroup analysis. BMI = body mass index, CI == confidence interval, HS = high school, OR = odds ratio, PIR = poverty–income ratio, W/D/S = widowed/divorced/separated.

### 3.4. Mediation analysis

Mediation analysis results demonstrated that BMI (obesity) had the most prominent mediating effect. The a-path (hysterectomy → BMI) OR was 1.33 (*P* < .001), the b-path (BMI → gallstones) OR was 2.52 (*P* < .001), the indirect effect was 0.055 (95% CI: 0.019–0.094), and the proportion mediated was 16.5% (*P* < .001). Hypertension also showed a significant mediating effect, with an a-path OR of 1.42 (*P* < .001), a b-path OR of 1.39 (*P* < .001), an indirect effect of 0.021 (95% CI: 0.007–0.038), and a proportion mediated of 6.5% (*P* = .004). The mediating effect of diabetes was also statistically significant, with an a-path OR of 1.71 (*P* < .001), a b-path OR of 1.76 (*P* < .001), an indirect effect of 0.046 (95% CI: 0.024–0.073), and a proportion mediated of 13.9% (*P* < .001). In contrast, hyperlipidemia did not show a significant mediating effect (indirect effect = 0.001, 95% CI: −0.004–0.007, *P* = .676), with a proportion mediated of only 0.3% (Fig. 3). These results indicate that BMI, hypertension, and diabetes partially mediate the association between hysterectomy and gallstones, while hyperlipidemia does not play a significant mediating role.

**Figure 3.**
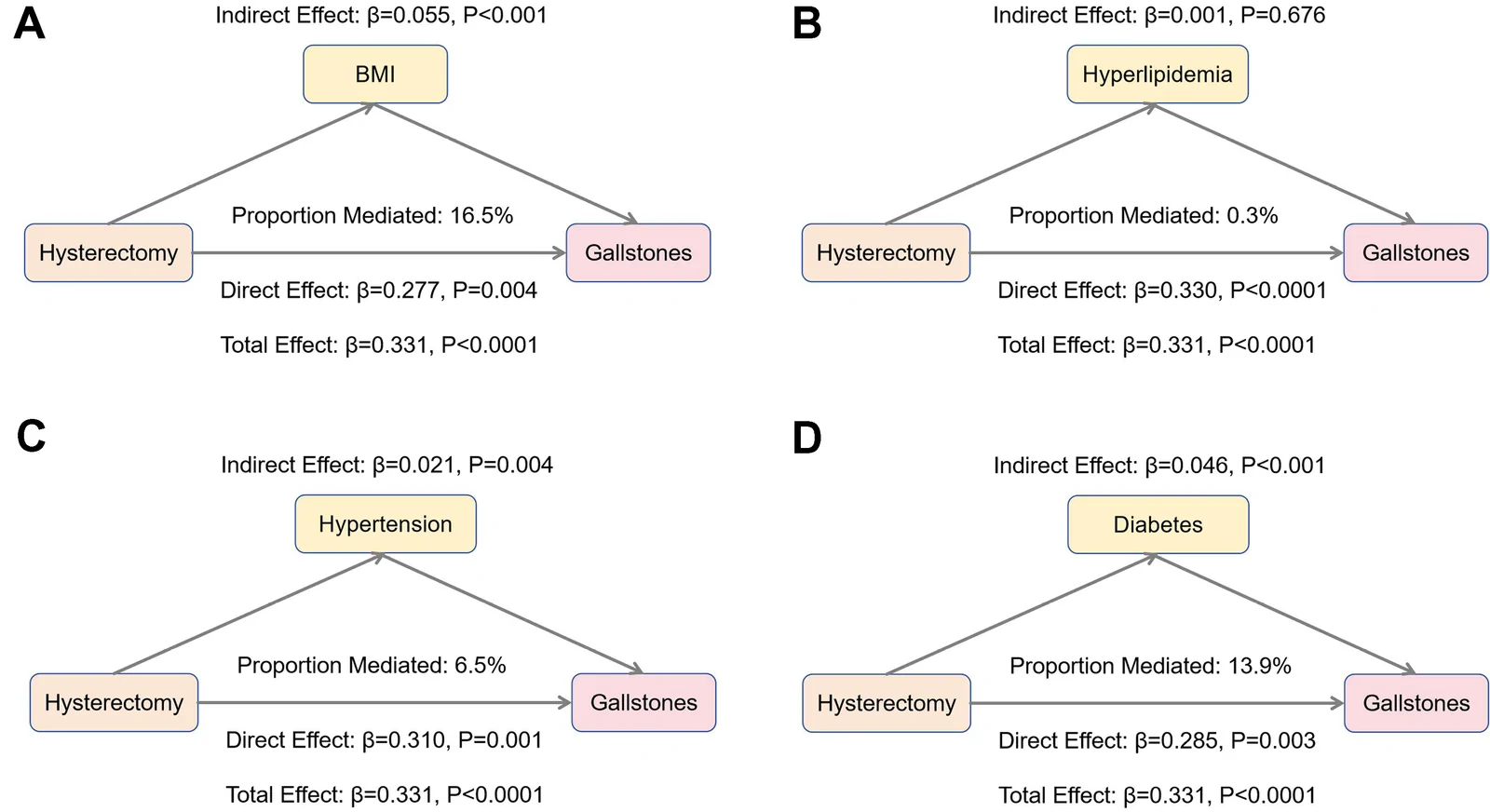
Mediation analysis. (A) BMI; (B) hyperlipidemia; (C) hypertension; (D) diabetes. BMI = body mass index.

## 4. Discussion

Utilizing nationally representative NHANES data, this study is the first to systematically investigate the epidemiological association between hysterectomy and gallstone risk in women, along with the underlying mediating mechanisms involving metabolic disorders. The main findings indicate that a history of hysterectomy remains significantly associated with a 32% increased risk of gallstones, even after comprehensive adjustment for a series of demographic, socioeconomic, lifestyle, and clinical confounding factors. More importantly, mediation analysis revealed that this association is not entirely direct but is partially transmitted through 3 metabolic intermediate phenotypes: BMI (obesity), hypertension, and diabetes. These findings provide a novel metabolic perspective for understanding the long-term, systemic health impacts of this common gynecological surgery and underscore the necessity for comprehensive metabolic management and biliary health monitoring in women post-hysterectomy.

Our core finding (that hysterectomy is an independent risk factor for gallstones) provides direct evidence for understanding the link between gynecological surgery and digestive system diseases. This association aligns with the clinical observation of a frequent co-occurrence of asymptomatic gallstones in hysterectomy patients.^[12]^ From a pathophysiological perspective, this link may be rooted in the shared foundation of metabolic disturbances between the 2 conditions. The identification of BMI, hypertension, and diabetes as significant mediators in this study robustly supports the “metabolic pathway” hypothesis. Hysterectomy may act as a triggering event, disrupting endocrine and metabolic homeostasis, thereby inducing or exacerbating obesity, glucose metabolism abnormalities, and elevated blood pressure.^[6–8]^ These metabolic disorders are classic risk factors promoting bile cholesterol supersaturation, gallbladder dysmotility, and stone formation.^[16]^ Our results are consistent with and integrate findings from previous studies: hysterectomy, even with ovarian conservation, is associated with a 32% increased risk of metabolic syndrome^[3]^; postoperative weight gain is common^[17]^; and hysterectomy is independently associated with elevated blood glucose and hypertension risk.^[6,7]^ Thus, our mediation analysis connects these disparate pieces of evidence, constructing a potential causal chain from “hysterectomy” to “metabolic abnormalities” and subsequently to “gallstones.”

Notably, BMI (obesity) was the factor with the strongest mediating effect. Obesity promotes gallstone formation through multiple mechanisms, including increased biliary cholesterol secretion, reduced bile acid pool, induction of insulin resistance, and chronic low-grade inflammation.^[18]^ This study showed that women with hysterectomy had a 33% increased risk of obesity, and obese individuals had 2.52 times the odds of gallstones compared to nonobese individuals. This finding highlights the paramount importance of postoperative weight management in preventing long-term complications. The mediating role of diabetes was also significant, and its mechanism may involve hyperinsulinemia affecting hepatic cholesterol metabolism and altering bile composition.^[19]^ Although the proportion mediated by hypertension was relatively smaller, it suggests an overall clustering of cardiometabolic risks. Collectively, these 3 factors mediated approximately 36.9% of the total effect, indicating that metabolic factors play a non-negligible role in the hysterectomy–gallstone association.

A finding warranting further discussion is the lack of a significant mediating effect for hyperlipidemia. This seems paradoxical to the conventional view that dyslipidemia is a key risk factor for gallstones. Possible explanations include: First, our definition of hyperlipidemia was based on a comprehensive lipid profile (ATP III criteria) rather than single cholesterol or TG metrics, which may mask the role of specific lipid components.^[20]^ Notably, hysterectomy may affect lipoprotein subfractions unevenly (e.g., high-density lipoprotein vs low-density lipoprotein vs TGs), such that a composite ATP III label could dilute component-specific mediating effects.^[21]^ Second, the impact of hysterectomy on the lipid profile may be complex, with varying changes in different lipoprotein components,^[22]^ and its net effect on gallstone risk might be less direct than that of obesity or diabetes. Third, the independent contribution of hyperlipidemia may be attenuated after adjusting for strongly correlated factors like obesity and diabetes. This negative result suggests that disturbances in energy metabolism and vascular regulation may play more central roles than pure lipid metabolism disorders in the metabolic mechanisms linking hysterectomy to gallstone risk.

Subgroup analysis revealed heterogeneity in this association across populations, with a stronger association particularly among women of lower socioeconomic status (PIR < 1.3). This finding may be related to factors such as accessibility to healthcare resources, health literacy, postoperative recovery conditions, and cumulative life stress,^[23]^ indicating a particularly urgent need for health interventions targeting disadvantaged groups. E-value analysis demonstrated that a relatively strong unmeasured confounder would be required to completely negate our primary findings, enhancing the credibility of the results.

This study has important clinical and public health implications. First, for gynecologists and general practitioners, women undergoing hysterectomy should be considered a potential high-risk group for gallstones. Preoperative evaluation could consider including abdominal ultrasound as a routine screening to detect asymptomatic stones. Second, long-term postoperative follow-up should not be limited to pelvic health but should extend to comprehensive metabolic monitoring, including regular assessment of BMI, weight, blood pressure, blood glucose, and lipids at 6 months, 12 months, and annually thereafter. Active lifestyle interventions are crucial for weight control and the prevention of diabetes and hypertension, which may not only benefit cardiovascular health but also directly reduce gallstone risk. Finally, for policymakers, consideration should be given to integrating the metabolic health management of post-hysterectomy women into broader chronic disease prevention and control systems.

This study has several limitations. First, the cross-sectional design cannot establish the temporal sequence or causal direction between hysterectomy and gallstones, a fundamental constraint that we wish to emphasize more strongly here. Although we logically infer surgery precedes stone formation, the possibility of reverse causality or residual confounding remains. Second, detailed information on hysterectomy indications, type (total vs subtotal, laparoscopic vs abdominal), surgical approach, oophorectomy status, the timing of hysterectomy relative to gallstone diagnosis, and menopausal status is limited in NHANES. Notably, we were unable to account for confounding by indication (e.g., uterine fibroids, endometriosis, abnormal uterine bleeding), which may independently relate to metabolic risk and GSD. These factors could significantly modify their association with metabolic outcomes and gallstones. Third, both hysterectomy status and gallstone diagnosis were based on self-report, which, although previously validated in NHANES-based studies, remains subject to largely non-differential misclassification bias that would more likely attenuate (bias toward the null) than inflate the observed association. Fourth, mediation analysis based on observational data infers “effects” in a statistical sense; biological experiments and longitudinal studies are needed to further validate the underlying mechanisms.

## 5. Conclusion

In conclusion, this large-scale nationally representative study found that hysterectomy is significantly associated with an increased risk of gallstones in women, and this association is partially mediated by post-hysterectomy obesity, hypertension, and diabetes. These findings link common gynecological surgery to an important digestive system disease via a metabolic bridge, deepening our understanding of the long-term health impacts of hysterectomy. It calls upon clinicians to pay greater attention to the long-term metabolic health and disease prevention of patients beyond the surgery itself. Future prospective cohort studies and mechanistic research are needed to confirm the causal direction of this association and elucidate the precise biological pathways involved.

## Author contributions

**Conceptualization:** Lang Song.

**Formal analysis:** Xiaolin Li.

**Investigation:** Xiaolin Li.

**Methodology:** Xiaolin Li.

**Software:** Xiaolin Li.

**Writing – original draft:** Xiaolin Li.

**Writing – review & editing:** Lang Song.
